# Use of Omalizumab to treat a nine-year old, with steroid-dependent, allergic asthma, adrenal insufficiency and vertebral compression fractures due to steroid induced severe osteoporosis

**DOI:** 10.1186/1710-1492-7-S2-A38

**Published:** 2011-11-14

**Authors:** Alicia Ring, Caroline Rizk, Stephanie Santucci, Joanne Desormeaux, Ian MacLuskey, Jacob Karsh, William H Yang

**Affiliations:** 1 Allergy and Asthma Research Centre, Ottawa, Ontario, Canada, K1Y 4G2; 2 University of Ottawa Medical School, Ottawa, Ontario, Canada

## Background

In Canada, Omalizumab is indicated for adults and adolescents with moderate to severe persistent allergic asthma, but not for pediatric use (<12 years of age). A 9 year-old boy with steroid dependent, allergic asthma, multiple ICU admissions and severe back pain from compression fractures was referred to our centre. IgE was 1337 IU/ml. Skin prick testing showed multiple positive reactions. Asthma treatment included inhaled corticosteroids and frequent courses of oral prednisone.

## Methods

After obtaining necessary approvals and informed consents, Omalizumab treatment, 375mg every 2 weeks, was initiated in September 2010. Serum cortisol levels, bone density, spirometry, and PAQLQ were used to monitor clinical response.

## Results

After 11 months, the changes below were noted.

**Table 1:** 

	Daily Prednisone	Daily ICS dose	FEV1	Serum cortisol^‡^	Bone density^*^	PAQLQ^†^
2010^a^	30 mg	800 mcg	1.46 L	12 nmol/L	0.626 g/cm^2^	4.7
2011 ^b^	NIL	200 mcg	1.98 L	215 nmol/L	0.686 g/cm^2^	7

^a^ Pre Omalizumab treatment

^b^ Post Omalizumab treatment

^‡^Morning results, range 185 – 624 nmol/L

^*^Total hip

^†^Pediatric asthma quality of life questionnaire with standardized activities: low 1 – 7 high

## Conclusions

The patient improved and was off oral/inhaled corticosteroids with no asthma exacerbations. Spirometry, serum cortisol, PAQLQ and bone density improved. Prednisone treatment in young asthmatic children can be associated with serious side effects. Omalizumab therapy can permit steroid withdrawal and resolution of side effects.

